# Emotion recognition using spectral-spatial attention multi-temporal scale network: EEG study

**DOI:** 10.1186/s40708-026-00309-x

**Published:** 2026-06-03

**Authors:** Zhe Tao, Guanghao Huang, Leilei Ma, Zhuochao Chen, Yinhua Liu, Keum Shik Hong

**Affiliations:** 1 Institute for Future, School of Automation, Qingdao University, Qingdao, 266071 China; 2 School of Mechanical Engineering, Pusan National University, Busan, 46241 Korea; 3 Shenzhen Aibisheng Technology Co., Ltd., Shenzhen, 518081 China

**Keywords:** Emotion recognition, Electroencephalogram (EEG), Multi-temporal scale, Spatio-temporal modeling, Attention mechanism

## Abstract

Electroencephalography (EEG) provides a non-invasive, portable, and cost-effective solution for emotion classification, but existing methods often struggle to capture spatial-spectral dependencies and multi-temporal scale dynamics inherent in EEG signals. This paper proposes a spectral-spatial attention multi-temporal scale network (SSA-MTSNet) to address these challenges. The SSA-MTSNet integrates 3 components: a spectral-spatial attention module, a multi-temporal scale spatio-temporal convolution module, and a long short-term memory (LSTM) module. First, the SSA-MTSNet preserves electrode topology and simultaneously enhances EEG signal frequencies and interactions among brain regions through attention mechanisms. Then, short- and long-term emotional cues are captured using multi-temporal scale convolution, followed by sequence modeling with LSTM. Evaluated on the SEED and SEED-IV datasets, the SSA-MTSNet achieves average accuracies of 98.34% and 91.79% respectively, and 95.13% and 95.30% on the valence and arousal classification tasks of DEAP, respectively. These results suggest that the proposed model achieves competitive performance across different experimental paradigms and emotion labeling strategies by jointly modeling complementary spectral, spatial, and temporal EEG information for emotion recognition.

## Introduction

Affective computing [1], a relatively new interdisciplinary field, investigates how computational systems can effectively process, recognize, and interpret human emotional expressions, thereby enabling more natural human–computer interaction (HCI) [2]. As a vital subdomain of affective computing, emotion recognition shows considerable potential in various application areas, including emotional healthcare, intelligent HCI systems, and personalized multimedia content recommendation [3]. A growing body of impactful and practical studies has emerged in this domain, such as emotion assessment for patients with disorders of consciousness (DoC) [4], and computer-aided diagnosis of autism spectrum disorder [5], attention-deficit/hyperactivity disorder (ADHD) [6], and depression [7, 8], as well as driver mood detection [9] and diagnosis of cognitive function [10–14]

Emotion, as a complex mental state, manifests in both physical behaviors and physiological responses. Its recognition primarily relies on information such as facial expressions, body movements, and physiological signals acquired via sensors [14]. Among these approaches, methods that achieve emotion recognition by capturing facial expressions and body movements [15, 16] can intuitively reflect emotional states, but they are susceptible to interference from voluntary masking or suppression by individuals, which may significantly reduce the accuracy of emotion recognition. In contrast, physiological signals, especially electroencephalography (EEG) signals, can accurately reflect changes in brain function across different brain regions [17–19] and, beyond that, provide a more objective and stable representation of internal emotional processes [20]. Related studies indicate that brain regions such as the amygdala and prefrontal cortex are critically involved in emotion regulation and processing [21, 22].

EEG has gained increasing attention among various physiological measures due to its noninvasive nature, affordability, and high temporal resolution [23, 24], making it particularly well-suited for tracking neural responses to emotional stimuli. Consequently, EEG-based emotion recognition has become a significant research focus in affective computing and neuroscience. In the early exploration of this field, conventional EEG-based machine learning approaches have long been a mainstream technical route, and their emotion classification process typically involves two core steps: manual feature extraction and classification algorithms. Features are commonly extracted from the time, frequency, or time–frequency domains. Time-domain features include higher-order crossings (HOC) [25] and Hjorth parameters [26], while frequency-domain features often rely on power spectral density (PSD) analysis [27]. In time–frequency analysis, the discrete wavelet transform (DWT) is widely used to extract fine-grained temporal and spectral features from EEG signals [28]. Additionally, entropy-based features have been extensively studied to capture the complexity and irregularity of neural dynamics. Representative examples include fractal dimension [27], approximate entropy [29], sample entropy [30], and differential entropy [31], with the latter being particularly favored for its robustness.

For classification, numerous algorithms have been adopted, including support vector machines (SVM) [32], k-nearest neighbors (KNN) [28], bayesian classifiers [33], random forests [34], perceptrons [35], and clustering techniques [36]. However, these conventional machine learning methods rely heavily on manually engineered features and shallow classifiers, making it difficult to capture the inherent richness, non-stationarity, and high-dimensional structure of EEG signals. When confronted with more complex emotion recognition tasks, this limitation becomes particularly pronounced, further driving the development of deep learning algorithms.

With the continuous advancement and performance improvements in deep learning algorithms, EEG-based emotion recognition methods have also made notable progress. A deep belief network (DBN) using handcrafted features [37], a channel-frequency convolutional neural network (CFCNN) based on recurrence quantification analysis [38], and a hierarchical convolutional neural network (HCNN) utilizing 2D sparse maps of differential entropy features [39] have been demonstrated for emotion classification while preserving the spatial topology of the electrodes. In addition to the feature-based CNN approaches mentioned above, another class of methods leverages spatial information more effectively by representing EEG data as graphs. A dynamic graph convolutional neural network (DGCNN) [40] is demonstrated to capture spatial dependencies, comparing several handcrafted features, including differential entropy (DE), power spectral density (PSD), differential asymmetry (DASM), and rational asymmetry (RASM), across five frequency bands.

In recent years, EEG-based emotion recognition research has increasingly emphasized improving cross-subject generalization and modeling richer spatio-temporal dynamics. The multi-modal EEG NEO-FFI [41] via an attention layer strengthens the interaction between EEG features and personality factors through a trained attention mechanism, thereby highlighting the role of individual traits. The automated extraction approach [42] leverages the noise-assisted multivariate empirical mode decomposition (NA-MEMD) to extract narrow-band intrinsic mode functions (IMFs), and Hilbert spectral analysis is performed on IMFs to obtain the marginal Hilbert spectrum (MHS) for calculating the spectral energy and yielding a spectral-temporal feature representation. The Tsallis entropy method [43] extracts discriminative sub-band features from multichannel EEG and combines them with KNN to enhance cross-subject recognition. The emotion preceptor model [44] introduces a static spatial adapter and causal temporal mechanisms to reduce inter-individual variability and achieve high-precision unimodal emotion recognition. The temporal-spectral graph convolutional network (TSGCN) [45] integrates BiLSTM and dynamic graph convolution to jointly capture temporal-spectral and spatial representations, while minimum category confusion and deep and shallow feature dynamic adversarial learning mechanisms mitigate label noise and domain mismatch.

Despite these advances, EEG-based emotion recognition still faces several critical challenges that warrant further investigation. First, EEG signals are treated as independent time series across individual channels, neglecting the intrinsic spatial dependencies among electrode sites. Given that emotions are associated with large-scale neural dynamics across distributed brain regions, preserving the spatial topology of EEG electrodes is essential for capturing meaningful inter-channel relationships. Second, CNN-based approaches rely on two-dimensional convolutions, which are often inadequate for jointly modeling the spatio-temporal characteristics of EEG signals. Third, temporal dependencies play a vital role in emotion recognition, conventional methods frequently struggle to capture both short- and long-range temporal structures effectively. These limitations highlight the need for novel architectures that can more comprehensively exploit the EEG signal’s spatial, temporal, and spectral dimensions in an integrated manner.

To address the challenges above, this paper proposes a serial spectral–spatial attention multi-temporal scale network (SSA-MTSNet) for EEG-based emotion recognition. The proposed framework integrates spectral–spatial attention, multi-temporal scale convolutional encoding, and sequential temporal modeling into a unified architecture that jointly captures spatial topology, spectral characteristics, and temporal dynamics of EEG signals.

In particular, the proposed method was designed to address three specific limitations in existing EEG emotion recognition studies.

i) Insufficient preservation of electrode topology. To address this issue, we project EEG features onto a structured 2D electrode layout and organize them across frequency bands and time, enabling the model to preserve the original spatial topology while jointly representing spectral, spatial, and temporal information.

ii) Limited capability of conventional 2D CNN-based models to jointly capture spatial and temporal dependencies. To address this limitation, we employ multi-temporal scale 3D convolution to jointly model temporal evolution alongside spatially structured EEG representations. Unlike conventional 2D CNN-based methods that mainly operate on static spatial patterns, the proposed 3D convolutional design enables the network to extract discriminative spatio-temporal features directly from EEG representations organized across time, frequency bands, and electrode topology.

iii) Difficulty in modeling both short-range and long-range temporal dynamics within a unified framework. To address this challenge, the proposed framework decomposes temporal modeling into two coordinated stages. Specifically, multi-temporal scale convolutional branches with progressive fusion are used to capture local temporal dependencies at different temporal resolutions, while an LSTM is introduced after multi-scale convolutional encoding to model global temporal context. We would like to emphasize that this hierarchical temporal modeling strategy is a central design feature of the proposed method.

The proposed SSA-MTSNet has been evaluated on widely used datasets, SEED, SEED-IV and DEAP achieved competitive results in EEG-based emotion recognition. The remainder of this paper is organized as follows: Section 2 presents the datasets and details of theproposed model. Section 3 outlines the experimentalsetup and reports the results. Section 4 provides analysisand discussion. Section 5 concludes the paper bysummarizing the main contributions.

## Materials and methods

### Dataset

The proposed SSA-MTSNet model for emotion recognition was comprehensively validated through ablation experiments on the widely used on the SEED [32, 37], SEED-IV [46] datasets, while its generalization ability was further evaluated on the DEAP [47]. The details of datasets are shown in Table 1.

**Table 1:** Details of SEED, SEED-IV and DEAP datasets

Item	SEED	SEED-IV	DEAP
Number of subjects	15	15	32
Number of sessions	3	3	1
Number of trials	15	24	40
Each trial duration	4 min	2 min	1 min
Number of channels	62	62	32
Emotion category	3-class	4-class	Valence / Arousal

The SEED dataset consists of recordings from 15 participants (7 males and 8 females; mean age: 23.27 ± 2.37 years). Each subject completed three sessions on separate days, including 15 movie clips (approximately 4 min each) equally distributed across three emotion categories: positive, neutral, and negative. EEG signals were recorded using a 62-channel cap, down-sampled to 200 Hz, and filtered with a 0 to 75 Hz band-pass filter.

The SEED-IV dataset also includes 15 subjects, each participating in three independent sessions conducted on different days. Each session comprises 24 movie clips (around 2 min each), categorized into four emotional states: happy, sad, neutral, and fear. EEG recordings were collected using the same 62-channel setup, down-sampled to 200 Hz, and filtered to a 1–75 Hz band-pass.

The DEAP dataset is also a widely used dataset for EEG-based emotion recognition. It consists of recordings from 32 participants (16 males and 16 females). Each subject watched 40 one-minute music video clips and rated their emotional states on a scale from 1 to 9 in terms of valence, arousal, liking, and dominance. EEG signals were recorded using 32 channels according to the international 10/20 system, and the original signals were sampled at 512 Hz. Each trial consisted of a 3-s pre-trial baseline and a 60-s stimulus segment. In this work, the DEAP dataset was used to verify the generalization ability of the model.

### The framework for EEG-based emotion recognition

**Fig. 1:**
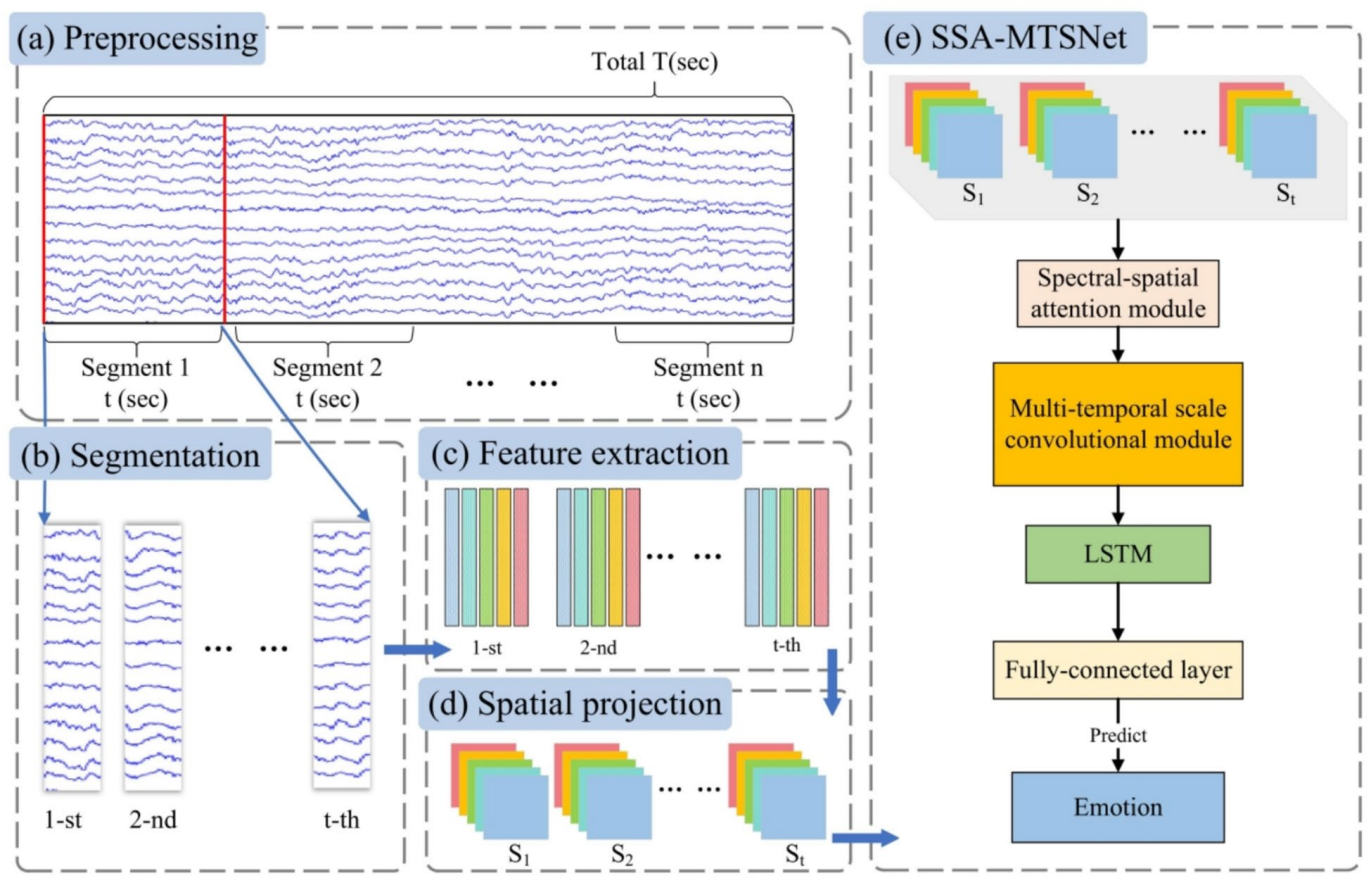
The overall framework for EEG-based emotion recognition using the spectral-spatial attention multi-temporal scale network (SSA-MTSNet): **a** EEG signal preprocessing includes signal denoising and an initial sample segmentation, **b** each segment from the initial segmentation is divided into *t* subsegments, **c** the calculated DE of five frequency bands in each subsegment, **d** spatial projection of features to the 2D channel layout matrix, and **e** SSA-MTSNet for the final emotional classification

The overall framework for the EEG-based emotion recognition using the proposed SSA-MTSNet is illustrated in Fig. 1. The left part of the framework outlines the data processing pipeline, including EEG signal acquisition, preprocessing and segmentation, feature extraction, and spatial projection. The right part presents the architecture of the proposed SSA-MTSNet, which consists of four core components: a spectral-spatial attention module, a multi-temporal scale spatio-temporal convolutional module, an LSTM layer for modeling global temporal dependencies, and a fully connected classifier for final emotion recognition.

The signal is first segmented into samples of length *t* seconds. Each sample is divided into the non-overlapping time slices of 1-s duration. Differential entropy (DE) features are then computed for each slice across five canonical frequency bands: *δ* (1–4 Hz), *θ* (4–8 Hz), *α* (8–13 Hz), *β* (13–30 Hz), and *γ* (30–50 Hz). EEG channels are projected into a two-dimensional matrix based on their relative spatial positions on the scalp to preserve the spatial correlations among electrodes. In the attention module, a serial spectral-spatial attention mechanism is incorporated to adaptively assign importance weights to different frequency bands and electrode locations. Next, a multi-temporal scale spatio-temporal convolutional module is employed to extract features at multiple temporal resolutions via multiple parallel branches. The outputs from all branches are fused and then passed into an LSTM layer, which captures long-range temporal dependencies across the entire EEG sequence. The hidden state from the final time step is used as the aggregated feature representation. Finally, a fully connected layer followed by a softmax activation is applied to generate the predicted probability distribution over emotional classes.

### Preprocessing and feature extraction

First, smoothing is applied to the raw EEG signals acquired from each electrode to reduce noise artifacts. Subsequently, the continuous EEG data from the SEED and SEED-IV datasets are segmented using a sliding window of length *t* seconds, with overlapping regions introduced to enhance sample diversity and temporal continuity. To maximize data utilization and prevent information loss, especially when the total signal duration is not an integer multiple of the window length, an overlap factor determines the percentage of overlap between adjacent windows, which is defined as:

1 $$ O = \frac{n \times t - T}{{\left( {n - 1} \right) \times t}}, $$

where $n = \left\lceil \frac{T}{t} \right\rceil$ denotes the number of windowed segments, *T* is the total duration of the signal, and *t* is the window length. Each segment of length *t* seconds is further divided into *t* non-overlapping 1-s slices. For each slice, differential entropy (DE) features are extracted from five EEG frequency bands of *δ*, *θ*, *α*, *β*, and *γ*. Assuming the EEG signal follows a Gaussian distribution $X\sim {\mathcal{N}}(\mu ,\sigma^{2} ),$ the differential entropy is defined as:

2 $$ \begin{aligned} DE(X) & = \int_{{ - \infty }}^{{ + \infty }} {\frac{1}{{\sqrt {2\pi \sigma ^{2} } }}} \exp \left( { - \frac{{(x - \mu )^{2} }}{{2\sigma ^{2} }}} \right) \\ & \quad \quad \log \left( {\frac{1}{{\sqrt {2\pi \sigma ^{2} } }}\exp \left( { - \frac{{(x - \mu )^{2} }}{{2\sigma ^{2} }}} \right)} \right)dx \\ & = \frac{1}{2}\log (2\pi e\sigma ^{2} ) \\ \end{aligned} $$

where *μ* and *σ*^*2*^ represent the mean and variance of the EEG segment, respectively, *e* and *π* are mathematical constants.

### Spatial mapping for DE features

**Fig. 2:**
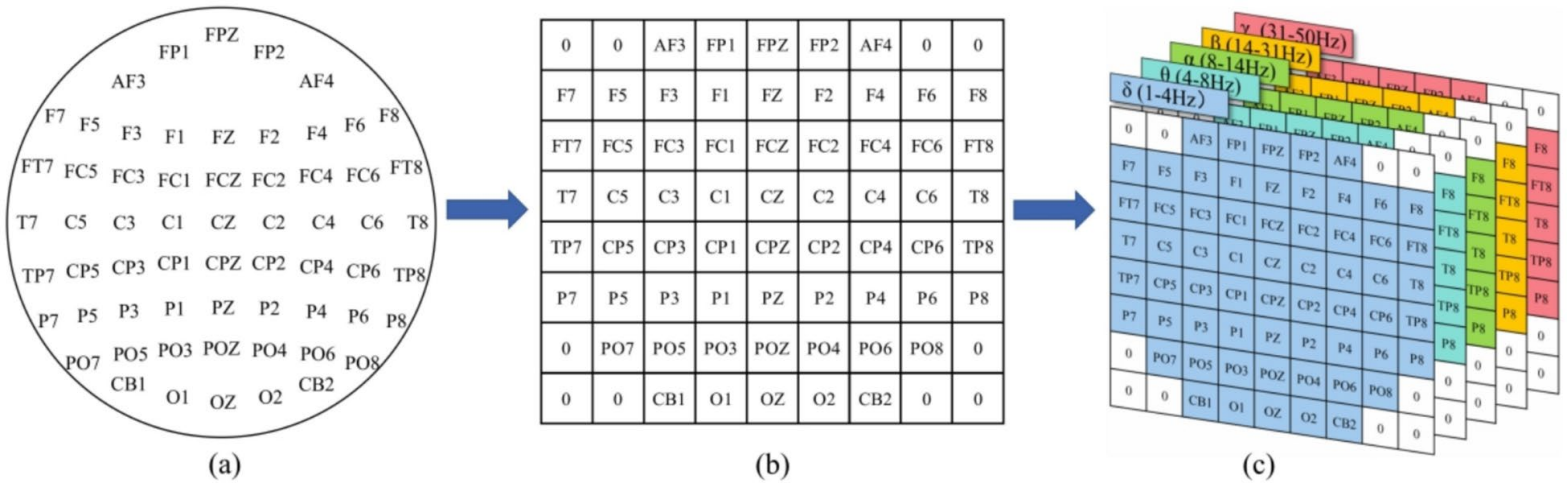
Spatial mapping and frequency band stacking: **a** The original electrode layout on the EEG cap, **b** the projected 2D electrode layout matrix based on the original electrode layout, and **c** DE features of five frequency bands; *δ* (1–4Hz), *θ* (4–8Hz), *α* (8–14Hz), *β* (14–31Hz), and *γ* (31–50Hz)

To capture the correlation of EEG signals across electrodes while preserving their relative spatial positions on the scalp, the differential entropy features extracted from the five frequency bands are mapped into a two-dimensional matrix and subsequently stacked, as shown in Fig. 2. Based on the original electrode layout shown in Fig. 2a, the 2D channel layout matrix is constructed as illustrated in Fig. 2b, where each electrode’s corresponding position is filled with feature values and the remaining positions are set to zero. Subsequently, the differential entropy features of each frequency band are mapped to a 2D channel layout matrix, as shown in Fig. 2c. Then, 2D matrices corresponding to the different bands are stacked along a new dimension to form a 3D structure with dimensions *B* × *H* × *W*, where *B* denotes the number of frequency bands, and *H*, *W* denotes the height and width of each 2D matrix. Finally, the 3D structures obtained at each second within the *t*-second EEG data are arranged in chronological order as a 4D structure $S = [S_{1} ,S_{2} , \ldots ,S_{t} ] \in {\mathbb{R}}^{{t \times {\mathrm{B}} \times {\mathrm{H}} \times {\mathrm{W}}}} .$

### The SSA-MTSNet

**Fig. 3:**
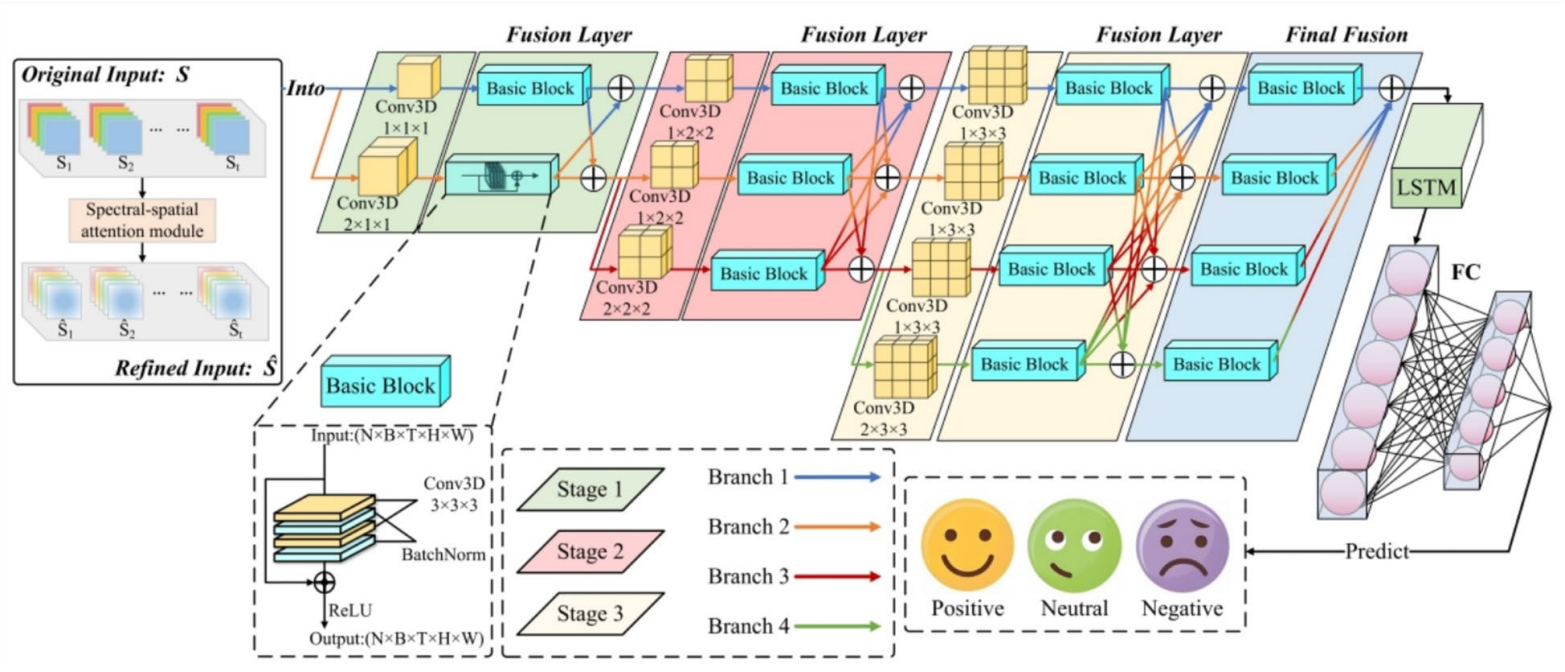
SSA-MTSNet’s architecture consisting of a spectral-spatial attention module, a multi-temporal scale convolutional module, an LSTM, and an FC layer for final emotional classification. The gradient lines represent the progressive fusion process in the fusion layer, where information is gradually transmitted and transformed from one branch to another during temporal alignment

The overall architecture of SSA-MTSNet is illustrated in Fig. 3. The proposed network consists of a serial spectral–spatial attention module, a multi-temporal scale convolutional module, an LSTM layer, and a fully connected (FC) classification layer.

The spectral-spatial attention module is applied to the input EEG representation to adaptively refine feature responses by sequentially emphasizing informative frequency bands and electrode regions.

Following the attention stage, a multi-temporal scale convolutional network is employed to extract spatio-temporal features from EEG segments. By introducing convolutional branches with different temporal receptive fields, the network captures local temporal dependencies at multiple resolutions and progressively constructs a richer representation that integrates both short-term and longer temporal dynamics.

After convolutional encoding, an LSTM layer is incorporated to further model temporal dependencies across the sequence. Positioned after the convolutional module, the LSTM operates on the extracted multi-scale representations and enhances the network’s capability to capture higher-level temporal context within EEG signals. Finally, the learned features are passed to a fully connected layer to perform emotion classification.

#### Spectral-spatial attention

**Fig. 4:**
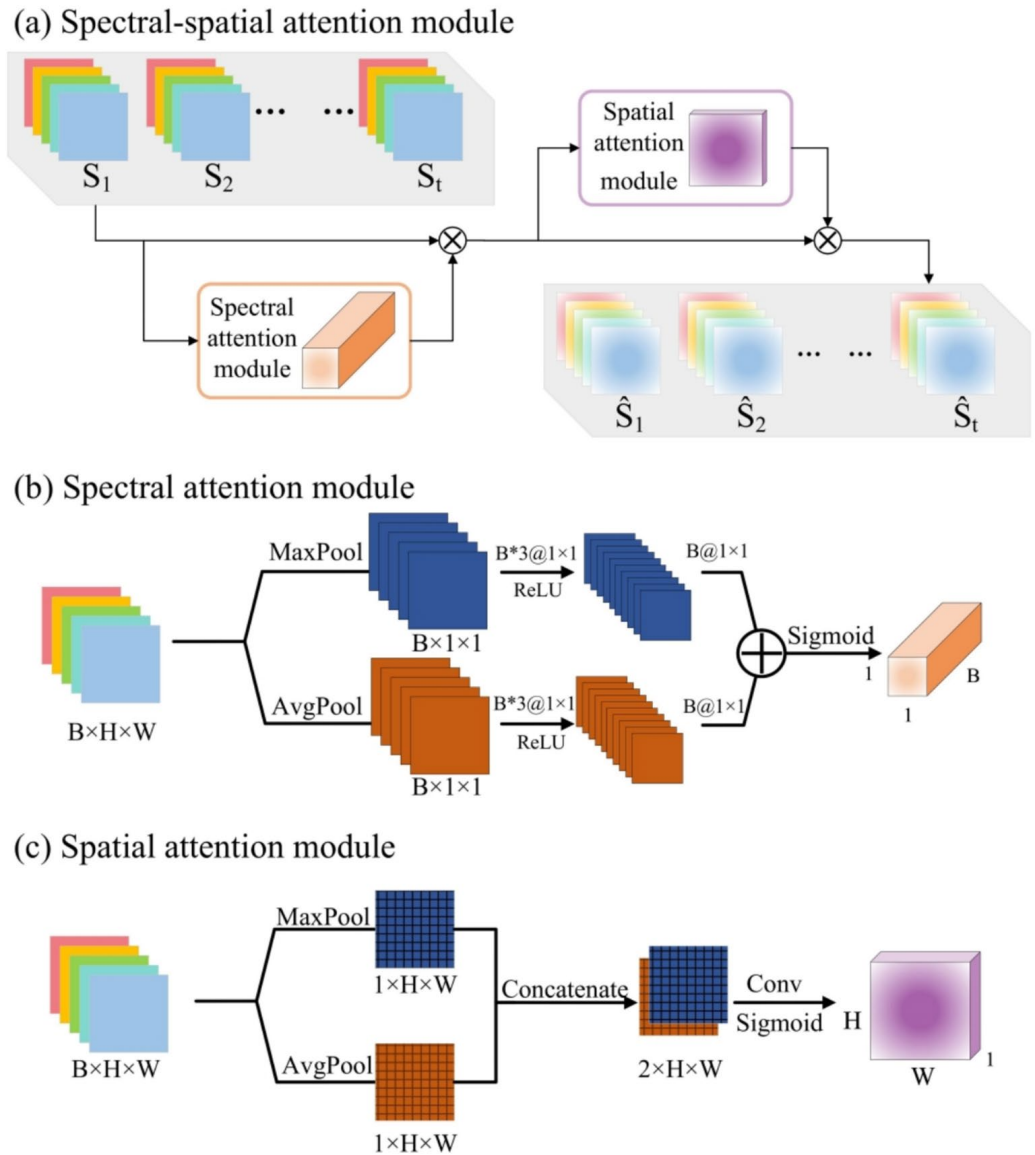
**a** The architecture of the spectral-spatial attention module consists of spectral attention module and spatial attention module, **b** the spectral attention module’s structure, and **c** the spatial attention module for generating spectral and spatial attention weights using max and average pooling

For each temporal segment $S_{i} \in {\mathbb{R}}^{{{\mathrm{B}} \times {\mathrm{H}} \times {\mathrm{W}}}} ,$ it is assumed that discriminative information exists across both the frequency and spatial domains. To emphasize the brain regions and frequency bands that play a decisive role in emotional processing, a dedicated attention module tailored for EEG-based emotion recognition is designed. Inspired by the convolutional block attention module (CBAM) [48], the proposed module adopts a serial architecture consisting of two submodules: a spectral attention module followed by a spatial attention module. The structure of the spectral-spatial attention module is illustrated in Fig. 4.

The spectral attention module is designed to emphasize informative frequency bands while suppressing less relevant ones. By dynamically adjusting the attention weights across frequency bands, this module helps the network focus on the most discriminative spectral components for emotion recognition. It achieves this by compressing the spatial dimension of the input feature map to compute frequency-specific attention weights.

To aggregate spatial information along the spectral dimension, the module applies two parallel operations, global average pooling and global max pooling, defined as:

3 $$ Z_{avg} \left( b \right) = \frac{1}{{{\mathrm{H}} \cdot {\mathrm{W}}}}\sum\limits_{i = 1}^{{\mathrm{H}}} {\sum\limits_{j = 1}^{{\mathrm{W}}} {S_{b,i,j} } } , $$

4 $$ Z_{max} \left( b \right) = \mathop {\max }\limits_{{i \in \left[ {1,2,...,{\mathrm{H}}} \right],j \in \left[ {1,2,...,{\mathrm{W}}} \right]}} S_{b,i,j} , $$

where $b \in [{1,2}, \ldots ,B],$ $Z_{avg} (b)$ and $Z_{max} (b)$ denote the average and maximum pooled values of the *b*-th frequency band, respectively. The pooled features are then passed through two sequential convolutional layers, each followed by a ReLU activation function. The outputs from both branches are summed and passed through a sigmoid activation to generate the spectral attention weights, defined as:

5 $$ F_{avg} (b) = {\mathrm{ReLU}}\left( {{\mathrm{W}}_{2}^{avg} \cdot {\mathrm{ReLU}}\left( {{\mathrm{W}}_{1}^{avg} \cdot {\mathrm{Z}}_{avg} (b)} \right)} \right), $$

6 $$ F_{max} (b) = {\mathrm{ReLU}}\left( {W_{2}^{max} \cdot {\mathrm{ReLU}}\left( {W_{1}^{max} \cdot Z_{max} (b)} \right)} \right), $$

7 $$ A_{spectral} = sigmoid\left( {F_{avg} \oplus F_{max} } \right), $$

where $W_{1}^{*} \in {\mathbb{R}}^{{{3} \times B}}$ and $W_{2}^{*} \in {\mathbb{R}}^{{B \times {3}}}$ are learnable parameters, $\oplus$ denotes element-wise addition, and $A_{spectral} \in {\mathbb{R}}^{B \times 1 \times 1}$ is the resulting spectral attention. Finally, the computed attention weights are applied to the input tensor *S* via element-wise multiplication to obtain the output of the spectral attention module:

8 $$ S^{spe} = A_{spectral} \cdot S. $$

The spatial attention module is designed to emphasize informative electrodes by adjusting the spatial attention weights across the electrode map. It increases the weights of features corresponding to important electrode positions while suppressing less relevant ones. This is achieved by compressing the frequency-band dimension to learn spatial-wise attention weights dynamically. Similar to the spectral attention module, this module employs two parallel branches using global average pooling and global max pooling to aggregate information across the frequency-band dimension, defined as:

9 $$ Z_{avg} (i,j) = \frac{1}{B}\sum\limits_{b = 1}^{B} {\left( {S_{b,i,j}^{spe} } \right)} , $$

10 $$ Z_{max} (i,j) = \max_{{b \in \{ 1,2, \ldots ,B\} }} \left( {S_{b,i,j}^{spe} } \right), $$

where $Z_{avg} (i,j)$ and $Z_{max} (i,j)$ represent the average and maximum pooled values across all frequency bands at spatial location (*i*,* j*), respectively. The pooled maps are then concatenated along the band dimension and passed through a convolutional layer followed by a sigmoid activation function to generate the spatial attention map, as expressed below:

11 $$ F = {\mathrm{ReLU}}\left( {W \cdot {\mathrm{Concat}}\left( {Z_{avg} ,Z_{max} } \right)} \right), $$

12 $$ A_{spatial} = sigmoid(F), $$

where ${\mathrm{Concat}} \left( {Z_{avg} ,Z_{max} } \right)$ denotes the concatenation along the band dimension, $W \in {\mathbb{R}}^{1 \times 2}$ is a learnable weight, and $A_{spatial} \in {\mathbb{R}}^{1 \times H \times W}$ is the resulting spatial attention map. The spatial attention is then applied to the spectrally attended feature map $S^{spe}$ to produce the refined *S* as follows:

13 $$ \hat{S} = A_{spatial} \cdot S^{spe} . $$

Finally, the refined feature representation $\hat{S} = \left[ {\hat{S}_{1} ,\hat{S}_{2} , \ldots ,\hat{S}_{t} } \right] \in {\mathbb{R}}^{t \times B \times H \times W}$ is obtained, in which both spectral and spatial attention mechanisms have been applied.

#### Multi-temporal scale convolutional network

**Fig. 5:**
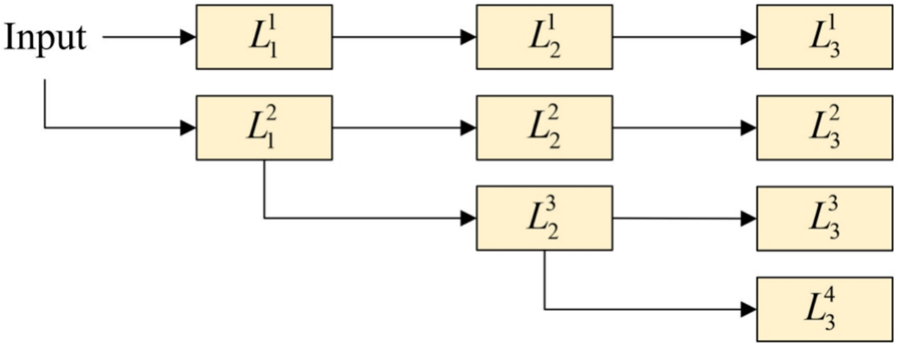
Parallel subnetworks in the multi-temporal scale convolutional network

**Fig. 6:**
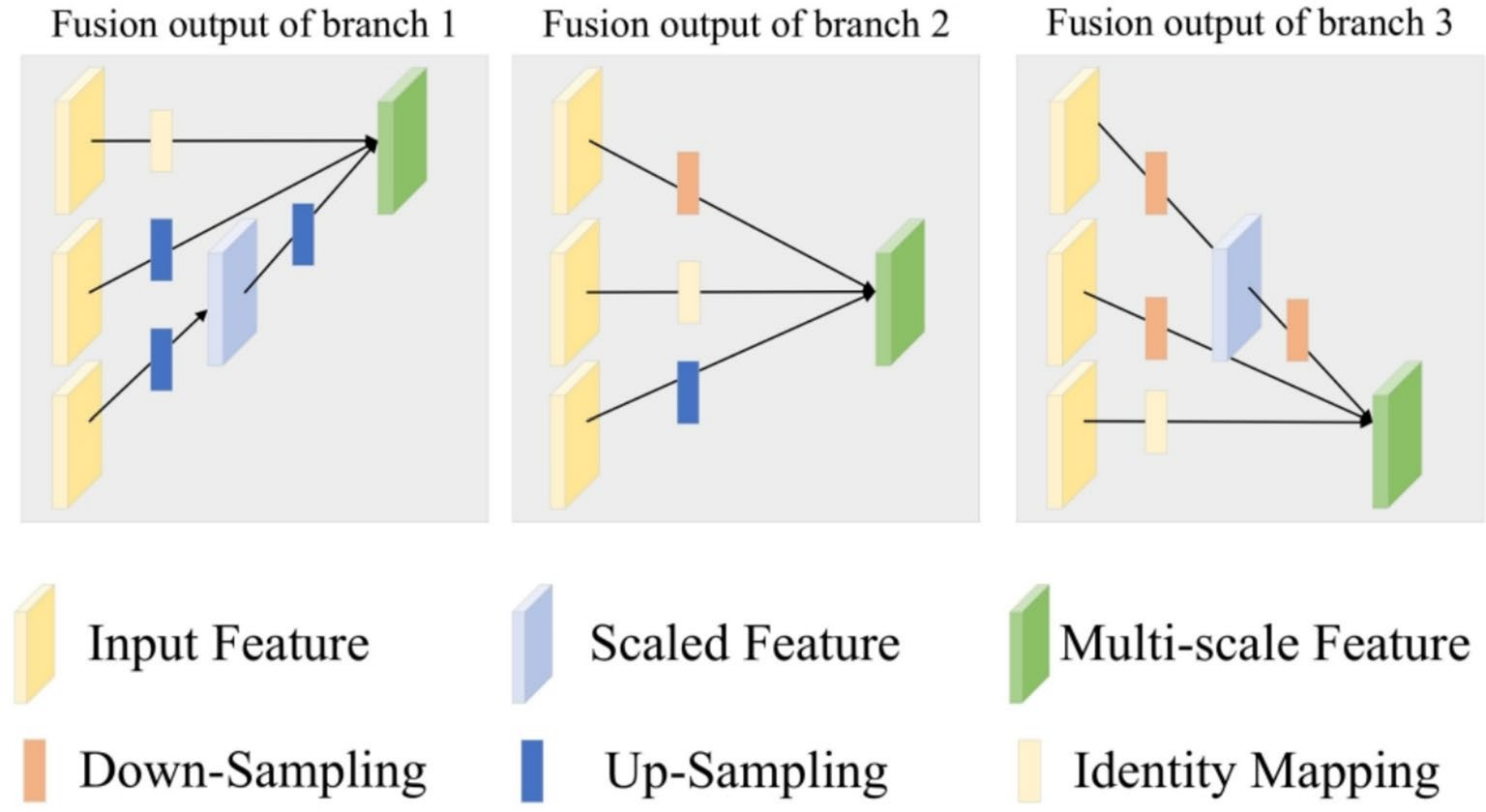
Illustration of the temporal alignment and feature fusion process among branches

The branches in the multi-temporal scale convolutional network are fused through dedicated fusion layers, enabling the network to capture local temporal dependencies at multiple resolutions and to construct a comprehensive representation that integrates both short-term and long-term patterns. Let $L_{s}^{n}$ denote the *n*-th branch at stage *s*, where different branches correspond to convolutional kernels with different temporal receptive fields, as shown in Fig. 5. The parallel branches at each stage operate at different temporal resolutions, allowing the network to model varying temporal dynamics. To enable information exchange across branches, a fusion layer is introduced to align feature representations with different temporal scales, as illustrated in Fig. 6.

**Algorithm 1:**
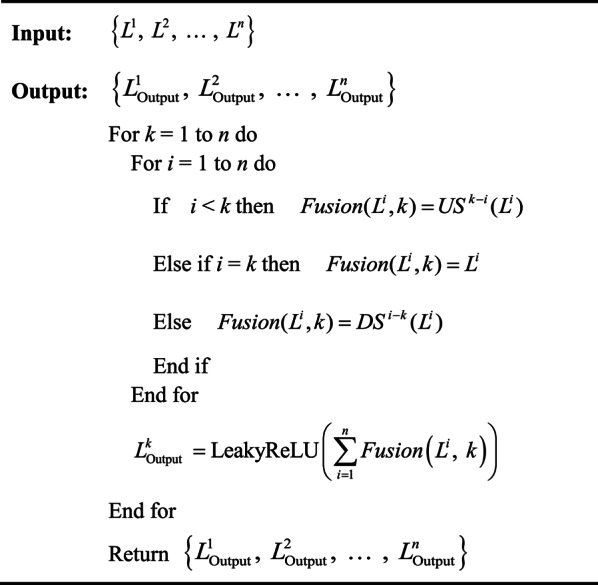
Progressive multi-temporal scale fusion process

To improve the readability of the fusion process, the progressive multi-temporal scale fusion procedure is summarized in Algorithm 1.

In Algorithm 1, $i,k \in \{ 1,2, \ldots ,n\}$, and $Fusion\left( {L^{i} , \, k} \right)$ denotes the alignment operation that transforms the feature map from the branch *i* to the temporal resolution of branch *k* through $|i - k|$ progressive up-sampling or down-sampling operations to transform the feature map. The operators *DS* and *US* denote single-step down-sampling using a 2 × 1 × 1 convolution and up-sampling using trilinear interpolation, respectively. Through this process, the fusion module aligns feature representations across different temporal scales and enables effective information exchange among branches.

Finally, the output feature maps from the multi-temporal scale convolutional network are flattened along the spatial and channel dimensions while preserving the temporal dimension and then passed into a single-layer LSTM network with a hidden size of 64. The LSTM models global temporal dependencies within the sequence and produces a temporally aggregated feature representation for subsequent classification.

## Experiments and results

Experiments were conducted on the SEED and SEED-IV datasets to evaluate the effectiveness of the proposed SSA-MTSNet. As described in Sect. 2, the input was represented as $S = [S_{1} ,S_{2} , \ldots ,S_{t} ] \in {\mathbb{R}}^{t \times B \times H \times W}$, the duration of each sample was set to 6 s, corresponding to the *t* in the input representation. As listed in Table 2, in the SEED dataset, 8595 samples of this 4D structure *S* can be obtained under session 1, session 2, and session 3, respectively, and in the SEED-IV dataset, the number of 4D structure samples *S* in the case of session 1, session 2, and session 3 is 8760, 8580, and 8475, respectively.

**Table 2:** Sample size in the SEED and SEED-IV datasets

Dataset	Session 1	Session 2	Session 3
SEED	8595	8595	8595
SEED-IV	8760	8580	8475

### Experiment setup

All experiments were conducted using tenfold cross-validation with strictly enforced subject-level data separation. After preprocessing, the EEG data were organized in the form: $(session,subject,segments,T,B,H,W)$, where session denotes the experimental session, subject represents individual participants, segments correspond to the segmented EEG samples derived from each subject, *T* denotes the temporal length of each sample, *B* represents the number of frequency bands, and *H* × *W* corresponds to the spatial electrode topology used to construct the 2D brain map representation. Then, tenfold cross-validation was performed at the subject level on the SEED and SEED-IV datasets. Specifically, subjects were first partitioned into ten folds, where each fold contained entire subjects. In each iteration, one fold of subjects was used for testing, while the remaining subjects were used for training.

After determining the training and testing subjects, the corresponding EEG segments were then reshaped into the form: $(session,segments,T,B,H,W)$ to construct the model input. This procedure ensures that all segments originating from the same subject remain entirely within either the training set or the testing set in each fold, thereby preventing potential cross-subject data leakage.

The proposed SSA-MTSNet contains 5.18 million trainable parameters and requires approximately 102.01 GFLOPs for a forward pass. The model occupies about 20.6 MB of parameter storage. All experiments were implemented in the PyTorch framework and trained on an NVIDIA RTX 4060Ti GPU using the Adam optimizer with a learning rate of 1e-4 and the cross-entropy loss function. Training was conducted for 60 epochs with a batch size of 64, and a dropout rate of 0.12 was applied to reduce overfitting. The training time is approximately 30–40 s per epoch, while the average inference time is 0.42 ms per sample.

**Fig. 7:**
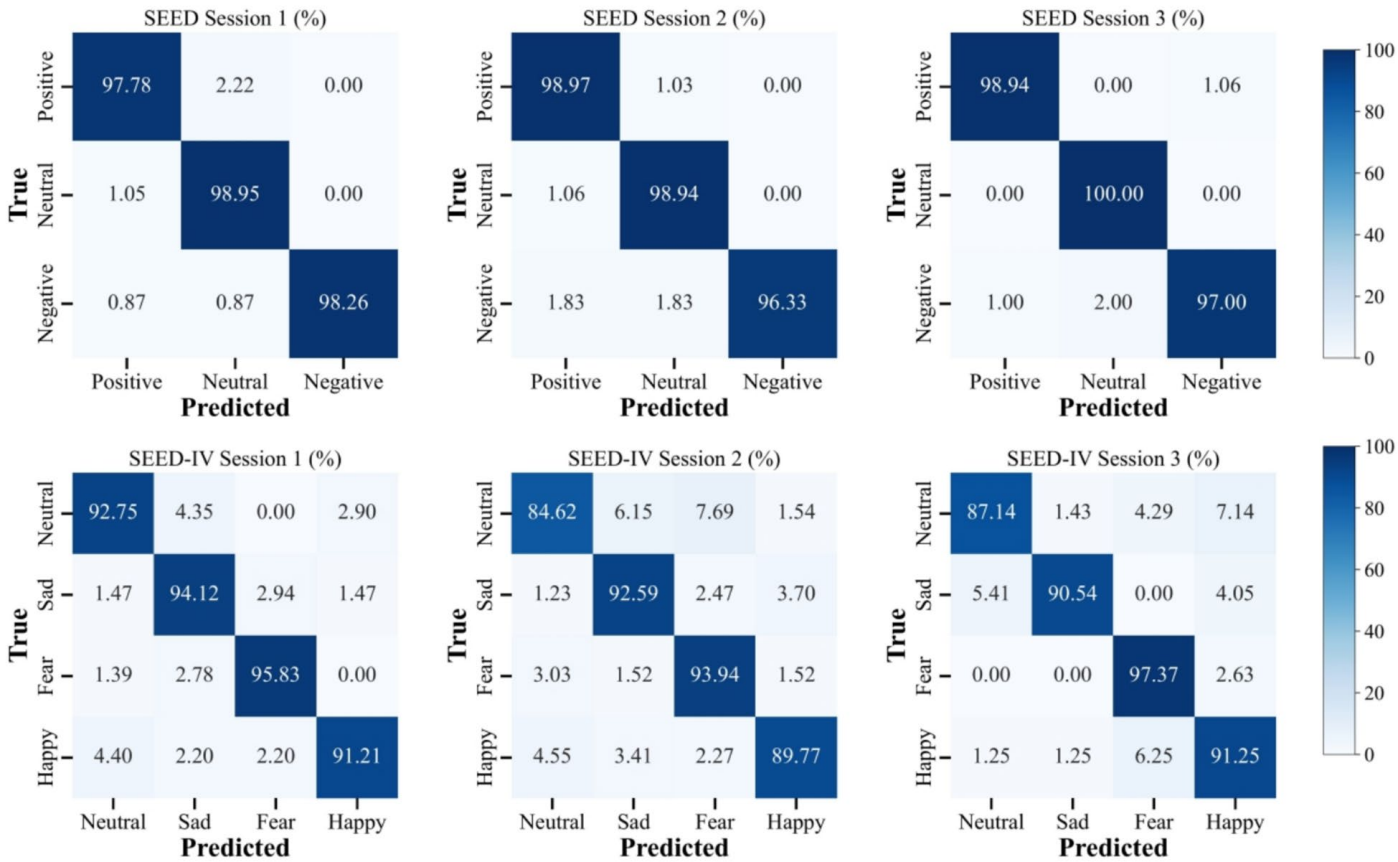
Confusion matrices on the SEED and SEED-IV datasets for each session using the proposed SSA-MTSNet

To further evaluate the reliability of the observed performance differences, paired t-tests were conducted on the fold-wise accuracies obtained under the tenfold cross-validation protocol, with the significance threshold set to *p* < 0.05. In addition, the confusion matrices of the proposed model on the SEED and SEED-IV datasets are presented in Fig. 7.

### Analysis of model performance under different combinations of attention

Experiments were conducted using different combinations of attention modules to evaluate their impact on the performance of the emotion recognition task. The following attention configurations were examined: no attention, spatial attention only, spectral attention only, a parallel combination of spatial and spectral attention, and serial combinations in two orders: the spatial-spectral attention (Spat.-Spec.) and spectral-spatial attention (Spec.-Spat.). All experiments were performed using the same data preprocessing procedures and training parameters.

The tenfold cross-validation accuracies and their corresponding standard deviations achieved by SSA-MTSNet under each attention configuration are summarized in Table 3. On both the SEED and SEED-IV datasets, the serial spectral-spatial attention configuration consistently yielded the best performance, with average accuracies of 98.34% and 91.79%, respectively. The spectral-spatial order significantly outperformed the spatial-spectral order in the two serial configurations. The results reveal that combining spectral and spatial features allows the model to capture feature patterns most efficiently, as marked in bold in Table 3.

**Table 3:** The performance of SSA-MTSNet under different attention configurations (Mean ± Standard deviation) (%)

	SEED				SEED-IV			
Configuration	Session 1	Session 2	Session 3	Avg acc	Session 1	Session 2	Session 3	Avg acc
No attention	96.15 ± 1.62	95.34 ± 1.72	96.92 ± 1.61	96.14	88.13 ± 2.05	89.28 ± 1.46	86.78 ± 2.04	88.06
Spatial only	95.73 ± 2.07	94.68 ± 2.57	95.45 ± 2.87	95.29	87.21 ± 3.07	88.69 ± 2.45	86.83 ± 3.58	87.58
Spectral only	96.74 ± 1.65	96.39 ± 1.77	97.09 ± 1.65	96.74	89.38 ± 2.23	90.32 ± 1.83	90.10 ± 2.10	89.93
Parallel	97.32 ± 1.74	96.34 ± 1.75	97.27 ± 1.96	96.98	90.75 ± 1.88	91.43 ± 1.55	90.33 ± 2.55	90.84
Spat.-Spec	96.40 ± 1.99	95.24 ± 1.85	95.80 ± 2.66	95.81	90.98 ± 2.76	89.83 ± 1.86	88.60 ± 3.35	89.80
**Spec.-Spat**	**98.18 ± 1.20**	**98.02 ± 2.03**	**98.83 ± 1.78**	**98.34**	**93.38 ± 1.66**	**90.47 ± 1.26**	**91.51 ± 1.84**	**91.79**

The parallel attention configuration also achieved strong results on both datasets, reaching 96.98% on SEED and 90.84% on SEED-IV, outperforming the single attention configuration. Moreover, using spectral attention alone resulted in relatively high performance, with 96.74% on SEED and 89.93% on SEED-IV. In contrast, the configuration utilizing only spatial attention exhibited the lowest accuracy across both datasets: 95.29% on the SEED dataset and 87.58% on the SEED-IV dataset. This result indicates that relying solely on spatial information not only barely improves classification performance but may even undermine the effectiveness of feature characterization, while further highlighting the critical role of spectral attention in enhancing feature separability.

To further examine the robustness of the results, paired t-tests were performed using the proposed Spec.-Spat. configuration as the reference model (Table 4). On the SEED dataset, the paired t-test results further show that the improvements of Spec.-Spat. over all other attention configurations are statistically significant (*p* < 0.05), confirming the effectiveness of the proposed attention design. On the SEED-IV dataset, although Spec.-Spat. still yields the highest average accuracy, the differences do not reach statistical significance. Therefore, the present results support the spectral-to-spatial order as a promising attention design, while further validation is still needed on more challenging datasets.

**Table 4:** Paired t-test results using the Spec.-Spat. configuration as the reference model

Configuration	SEED (t, p)	SEED-IV (t, p)
No-attention	t = 9.23, *p* = 0.011	t = 2.92, *p* = 0.10
Spatial-only	t = 10.07, *p* = 0.0097	t = 3.27, *p* = 0.08
Spectral-only	t = 18.30, *p* = 0.003	t = 1.64, *p* = 0.24
Parallel	t = 5.35, *p* = 0.033	t = 0.91, *p* = 0.46
Spat.-Spec	t = 6.63, *p* = 0.022	t = 2.88, *p* = 0.10

**Fig. 8:**
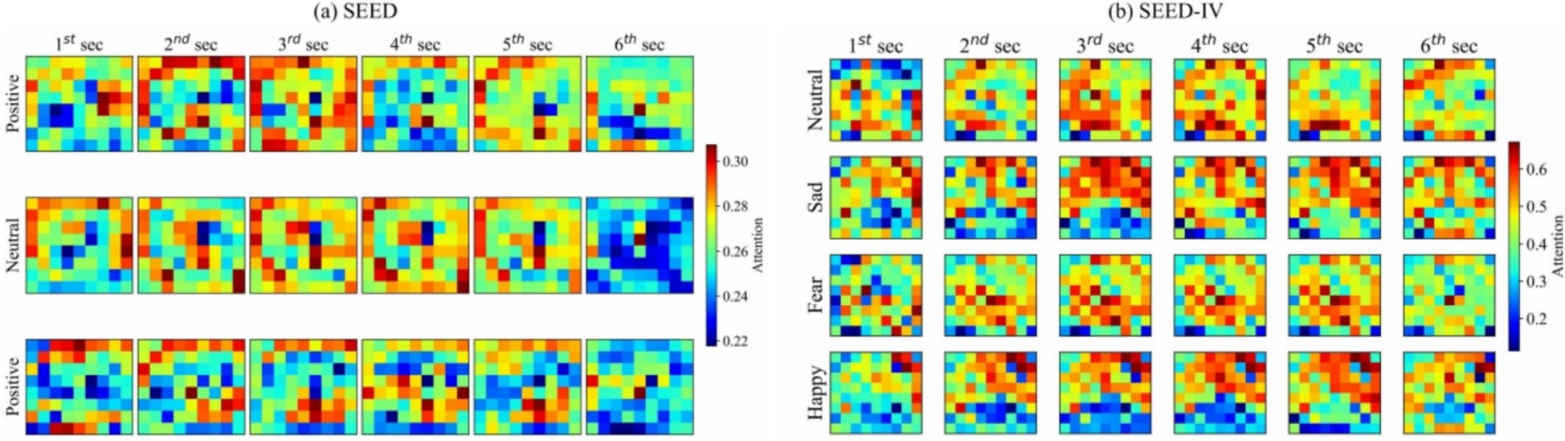
Spectral-spatial attention weights of SSA-MTSNet on the **a** SEED and **b** SEED-IV datasets

The attention weights extracted from the spectral-spatial attention module were projected onto the 2D channel layout matrix, as shown in Fig. 2b. In addition, Fig. 8 illustrates the temporal evolution of these weights across emotion categories in the SEED and SEED-IV datasets. Warmer colors indicate higher attention values, reflecting the relative importance of specific electrodes in emotion recognition decisions.

As shown in the attention visualization, electrodes with consistently high attention weights are concentrated in the prefrontal regions, including AF3, FP1, FPZ, FP2, and AF4, and in the lateral temporal regions, including F7, FT7, T7, TP7, F8, FT8, T8, and TP8. This spatial distribution is neurophysiologically meaningful, as the prefrontal cortex is known to be involved in emotion evaluation, cognitive control, and affective regulation, while temporal regions are associated with the integrating emotional and perceptual information, especially during affective stimulus processing. These findings are in line with previous studies in neurobiology and affective neuroscience [19, 49–52]. Furthermore, the visualization shows that the attention distribution changes over time, indicating the dynamic evolution of emotional states. This result demonstrates that SSA-MTSNet effectively captures temporally varying emotional patterns while emphasizing brain regions involved in emotion processing.

The concentration of attention weights in these regions suggests that the proposed SSA-MTSNet can focus on neural patterns plausibly related to emotional processing. This observation indicates that the spectral–spatial attention mechanism not only improves feature discrimination but also enhances the model’s neurophysiological interpretability. These findings further suggest that the proposed framework may provide useful insights for interpreting spatial neural patterns in EEG-based emotion recognition.

### Analysis of model performance under different branch and stage configurations

To comprehensively evaluate the contributions of each branch, stage module, and the fusion layer, ablation experiments were conducted using eight configurations under the same spectral-spatial attention mechanism, the results are summarized in Table 5.

**Table 5:** The performance of SSA-MTSNet with different branch and stage configurations (Mean ± Standard deviation) (%)

	SEED				SEED-IV			
Configuration	Session 1	Session 2	Session 3	Avg acc	Session 1	Session 2	Session 3	Avg acc
Branch 1	97.35 ± 2.04	96.22 ± 3.30	96.84 ± 3.17	96.80	90.34 ± 3.30	90.21 ± 2.35	88.46 ± 2.91	89.67
Branch 1&2	97.74 ± 1.05	96.57 ± 1.64	97.58 ± 0.97	97.30	91.14 ± 2.26	90.74 ± 1.82	88.92 ± 2.02	90.27
Branch 1&2&3	97.75 ± 1.01	96.65 ± 1.46	97.53 ± 1.10	97.31	88.79 ± 4.11	88.73 ± 3.00	86.99 ± 3.61	88.17
Stage 1 only	96.10 ± 1.15	95.06 ± 1.32	96.20 ± 0.80	95.79	82.60 ± 6.08	83.43 ± 4.42	80.68 ± 4.88	82.24
Stage 2 only	95.43 ± 1.51	93.05 ± 1.63	94.42 ± 2.02	94.30	82.52 ± 4.25	83.11 ± 3.13	81.45 ± 3.54	82.36
Stage 3 only	93.32 ± 2.03	91.80 ± 1.77	93.17 ± 1.87	92.76	77.16 ± 6.18	78.87 ± 5.44	76.50 ± 6.03	77.51
Stage 1&2	97.14 ± 1.11	96.07 ± 1.31	97.48 ± 0.52	96.90	90.68 ± 2.17	88.55 ± 2.54	87.17 ± 3.01	88.80
w/o fusion layer	94.31 ± 1.41	92.61 ± 2.08	93.50 ± 1.81	93.47	77.47 ± 3.47	80.97 ± 1.87	76.17 ± 2.44	78.20
**SSA-MTSNet**	**98.18 ± 1.20**	**98.02 ± 2.03**	**98.83 ± 1.78**	**98.34**	**93.38 ± 1.66**	**90.47 ± 1.26**	**91.51 ± 1.84**	**91.79**

These configurations included a single-branch model using only branch 1, a dual-branch model using branches 1 and 2, and a triple-branch model using branches 1 through 3. Additionally, models with different stage configurations were tested, including only stage 1, only stage 2, only stage 3, the combination of stage 1 and stage 2, and a configuration without the fusion layer. All of these configurations were compared with the complete SSA-MTSNet model (marked in bold in Table 5), which incorporates all branches, stages, and the fusion layer. Each model was trained and evaluated using tenfold cross-validation in each experiment to ensure consistency in comparison. All experiments were performed using the same data preprocessing procedures and training parameters.

The performance differences among models with different branch configurations are minimal. On the SEED dataset, increasing the number of branches from 1 to 3 led to steady improvements in average accuracy, rising from 96.80% with a single branch to 97.31% when using all three branches. Additionally, performance variability decreased, with the maximum standard deviation across sessions dropping from 3.30 to 1.46%.

In contrast, on the SEED-IV dataset, adding a third branch resulted in decreased performance. The average accuracy dropped from 90.27% using two branches to 88.17% with all three branches, while the maximum standard deviation across sessions increased from 2.26 to 4.11%. Notably, the complete SSA-MTSNet model overcomes this issue, achieving the highest average accuracy of 98.34% on the SEED dataset and 91.79% on the SEED-IV dataset, while maintaining low variability, with standard deviations of 1.20% and 1.26%, respectively. These results indicate that the best performance is achieved by the complete architecture, which combines multi-branch temporal modeling with progressive stage design and feature fusion. However, because the full model differs from the reduced variants in more than one component, the isolated contribution of branch number alone should not be over-interpreted.

Among single-stage variants, the model comprising only stage 1 performs best. On the SEED dataset, stage 1 alone achieves an average accuracy of 95.79%, compared with 94.30% for stage 2 and 92.76% for stage 3. A similar pattern is observed in the SEED-IV dataset, where stage 1 yields 82.24%, compared with 82.36% for stage 2 and 77.51% for stage 3. These results indicate that the SSA-MTSNet architecture’s hierarchical design, which progressively adds temporal scales, is more effective than models relying solely on multi-branch stages without a progressive mechanism. Furthermore, the performance gains observed in models containing stages 1 and 2, as well as in the SSA-MTSNet variant without a fusion layer, confirm that the absence of progressive branching hinders the fine-grained modeling of local temporal dependencies, ultimately leading to performance degradation.

Finally, a comparison between SSA-MTSNet models with and without the fusion layer highlights its critical role. The inclusion of the fusion layer significantly enhances performance, underscoring its importance in integrating multi-branch features. On SEED, the model without the fusion layer achieves an average accuracy of 93.47%, while the SSA-MTSNet reaches 98.34%, marking a significant improvement of 4.87%. On SEED-IV, the accuracy increases from 78.20 to 91.79%, a gain of 13.59%.

These improvements highlight the fusion layer’s role in effectively integrating multi-stage representations, thereby enhancing the model’s expressive power and its ability to capture both global and local temporal patterns. Paired t-tests were also conducted for the branch and stage ablation study, with the complete SSA-MTSNet taken as the reference model (Table 6). The paired t-test results further reveal that, on SEED, the proposed model significantly outperforms all single-stage variants as well as the model without the fusion layer (*p* < 0.05). A similar pattern is observed on SEED-IV, where significant improvements are also obtained over the single-stage settings and the variant without fusion. Although the differences between SSA-MTSNet and some branch configurations are not statistically significant, SSA-MTSNet still achieves the best overall performance. These results suggest that the progressive stage design and the fusion layer are particularly important to the effectiveness of the proposed architecture.

**Table 6:** Paired t-test results using SSA-MTSNet as the reference model

Configuration	SEED (t, p)	SEED-IV (t, p)
Branch-1	t = 4.28, *p* = 0.05	t = 2.28, *p* = 0.15
Branch-1&2	t = 3.38, *p* = 0.07	t = 1.68, *p* = 0.23
Branch-1&2&3	t = 3.42, *p* = 0.07	t = 3.85, *p* = 0.06
Stage-1 only	t = 9.97, *p* = 0.01	t = 7.61, *p* = 0.02
Stage 2 only	t = 6.07, *p* = 0.03	t = 8.90, *p* = 0.01
Stage 3 only	t = 14.14, *p* = 0.005	t = 10.32, *p* = 0.01
Stage 1&2	t = 5.41, *p* = 0.03	t = 4.19, *p* = 0.05
w/o fusion layer	t = 9.73, *p* = 0.01	t = 6.63, *p* = 0.02

## Discussion

In this paper, SSA-MTSNet was extensively evaluated on two public EEG emotion recognition datasets, SEED and SEED IV, and demonstrated consistently strong performance. Ablation studies are conducted to analyze the impact of attention configurations, branch combinations, and fusion strategies. The model achieves a high average accuracy of 98.34% on SEED and 91.79% on SEED IV when using the serial spectral-to-spatial attention configuration, confirming the benefit of sequential attention. In the branch configuration analysis, using three branches led to improved accuracy and reduced variability compared to a single-branch setting, with accuracy increasing from 96.80 to 97.31% and the maximum standard deviation decreasing from 3.30 to 1.46%. The fusion mechanism proved to be essential as well. Removing the fusion layer caused a clear performance drop, with average accuracy falling from 98.34 to 93.47% on SEED, and from 91.79 to 78.20% on SEED IV. These findings validate the effectiveness of the multi-stage spatio-temporal fusion design in aligning features and enhancing overall model robustness. The following section discusses the model’s performance across different input lengths, compares it with other representative methods, and further evaluates its generalization ability on the DEAP dataset.

### The effect of EEG segment length

The selection of EEG segment length is a critical factor in emotion recognition, as it directly influences the granularity of temporal features and the availability of contextual information. To investigate this effect, different segment lengths were evaluated using a model pre-trained on 6-s segments, as shown in Table 7.

**Table 7:** The performance of SSA-MTSNet with different EEG segment lengths. (Mean ± Standard deviation) (%)

	SEED				SEED-IV			
t(s)	Session 1	Session 2	Session 3	Avg acc	Session 1	Session 2	Session 3	Avg acc
4	83.24 ± 4.18	78.48 ± 6.62	84.62 ± 2.42	82.11	71.22 ± 6.39	64.01 ± 11.18	66.15 ± 6.56	67.13
5	96.18 ± 1.22	95.31 ± 1.69	96.14 ± 1.86	95.88	91.47 ± 1.56	90.13 ± 2.64	89.06 ± 1.64	90.22
6	98.18 ± 1.20	98.02 ± 2.03	98.83 ± 1.78	98.34	93.38 ± 1.66	90.47 ± 1.26	91.51 ± 1.84	91.79
7	98.07 ± 1.00	97.48 ± 1.41	97.91 ± 1.42	97.82	94.75 ± 1.58	93.89 ± 1.22	92.97 ± 1.38	93.87
8	97.92 ± 0.97	96.99 ± 1.37	97.67 ± 1.47	97.53	94.85 ± 1.60	93.85 ± 1.07	92.95 ± 1.41	93.88
9	98.01 ± 0.94	97.33 ± 1.24	97.93 ± 1.27	97.76	95.18 ± 1.23	94.14 ± 1.05	93.41 ± 1.23	94.24
10	97.93 ± 0.86	97.07 ± 1.31	97.79 ± 1.37	97.60	94.84 ± 1.54	94.02 ± 1.07	93.11 ± 1.20	93.99

 Experiments on models pre-trained with 6-s segments across varying paragraph lengths reveal that segment length choice has a substantial impact on emotion recognition performance. When using shorter segments and setting *t* to 4 s, model performance deteriorates significantly, with average accuracy dropping to 82.11% on SEED and 67.13% on SEED-IV. This degradation may stem from insufficient spatio-temporal context within the shortened window, which hinders the model’s ability to capture the full temporal dynamics of emotional evolution.

Extending the segment and setting *t* to 5 s leads to a substantial improvement, raising accuracy to 95.88% on SEED and 90.22% on SEED-IV, suggesting that even a modest increase in window length provides richer emotional cues. When *t* was set to 6 s, the model achieves relatively stable performance, attaining 98.34% on SEED and 91.79% on SEED-IV with reduced bias on SEED-IV. It indicates an optimal balance between temporal resolution and contextual richness, supporting both the sensitivity to short-term fluctuations and the representation of long-term emotions.

However, further increasing *t* beyond 6 s results in declining performance on SEED, while SEED-IV continues to show modest gains. After *t* was exceed 8 s, the improvement became negligible, indicating that although longer windows offer more contextual information for complex emotion recognition, they also introduce noise. It can cause the model to rely excessively on global averages, thereby diminishing its responsiveness to transient emotional variations.

These findings not only highlight the strong generalizability of SSA-MTSNet across varying segment lengths but also offer deeper insight into the benefits of multi-temporal scale modeling for EEG-based emotion recognition. Emotional states evolve dynamically over time, and the associated EEG patterns are likely to encode discriminative information across multiple temporal scales. Shorter segments are more sensitive to rapid fluctuations in emotional states, whereas longer segments provide richer contextual information related to more sustained affective processes. Consequently, relying on a single temporal scale may be insufficient to fully capture the complexity of emotional dynamics. On the SEED dataset, the best average accuracy was obtained with 6-s segments, whereas on SEED-IV the highest average accuracy was observed with 9-s segments. Therefore, 6 s should be regarded as a practical segment length used in the main experiments rather than a universally optimal choice across datasets.

### Compared with existing methods

To demonstrate the effectiveness of the proposed method, comparisons were conducted with several representative approaches listed below, including both classical and state-of-the-art models for EEG-based emotion recognition. These approaches employ a range of modeling strategies, such as spatio-temporal fusion, graph learning, attention mechanisms, and transformer-based architectures.

Although the core ideas and overall architectures of these baseline models can be approximately reconstructed from the published descriptions, their reported results cannot be strictly reproduced, mainly because some studies do not provide sufficiently detailed information on data preprocessing, feature extraction, or training settings. As a result, independent implementations may introduce unintended performance variations due to differences in experimental details. To avoid such potential bias and ensure a fair comparison, the baseline results reported in Table 6 were directly cited from the original publications.

i) 4D-aNN [53] constructs a 4D spatial-spectral-temporal representation of EEG signals and incorporates spatial and spectral attention mechanisms, which are consistent with ours. After extracting spatial and spectral features using CNN, it employs Bi-LSTM to model temporal dependencies and integrates attention mechanisms across the three-dimensional structure.
ii) MDGCN-SRCNN [54] combines a multi-layer dynamic graph convolutional network (MDGCN) with a style-based recalibration convolutional network (SRCNN). A style recalibration module is introduced into the CNN to enhance emotion-related features. By explicitly modeling inter-channel connectivity and style-based feature selection, the model fuses shallow spatial structures with deep semantic abstractions to achieve multi-scale feature integration.
iii) DBGC-AFFNet-AFTL [55] proposes a dual-branch dynamic graph convolutional network, combined with an adaptive transformer-based feature fusion module, supporting subject-independent transfer learning. It separately models DE and PSD features and utilizes multi-head attention and channel reweighting for high-level feature integration. The adapter-based fine-tuning strategy enables efficient cross-subject generalization.
iv) ACTNN [56] adopts a parallel spatial-spectral attention mechanism to enhance the salient regions and frequency bands in each time slice. It combines convolutional and transformer modules to extract multi-domain information. The transformer module employs multi-head attention to capture global temporal dependencies, demonstrating a strong capability in spatial-spectral-temporal feature fusion.
v) Bi-ViTNet [57] is based on the vision transformer framework. It consists of two branches, built with linear embeddings and transformer encoders, designed to model spatio-temporal and spatial-frequency features, respectively.
vi) AMDET [58] employs multi-dimensional EEG features and multi-head self-attention to model cross-domain dependencies. Spectral-spatial transformer layers extract informative representations, while a temporal attention layer emphasizes salient time frames.

As shown in Table 8, although the accuracy of SSA-MTSNet (marked in bold in Table 8) is slightly lower than that of the top-ranked ACTNN model on the SEED and SEED-IV datasets, the proposed method still demonstrates competitive overall performance. Notably, the stability of SSA-MTSNet is higher than that of ACTNN on both datasets, and it achieves the highest stability among all compared methods on SEED-IV. This characteristic is particularly desirable in EEG-based emotion recognition, where inter-subject variability and signal noise often lead to substantial performance fluctuations across test samples.

**Table 8:** Comparison with existing methods on the SEED and SEED-IV datasets

Methods	SEED	SEED-IV
	Acc ± Std (%)	Acc ± Std (%)
4D-aNN [53]	96.25 ± 1.86	86.77 ± 7.29
MDGCN-SRCNN [54]	95.08 ± 6.12	85.52 ± 11.58
DBGC-AFFNet-AFTL [55]	97.31 ± 1.47	89.97 ± 2.85
ACTNN [56]	98.47 ± 1.72	91.90 ± 5.43
Bi-ViTNet [57]	97.55 ± 1.58	88.08 ± 6.32
AMDET [58]	97.17 ± 0.93	87.32 ± 1.79
**SSA-MTSNet (This work)**	**98.34 ± 1.67**	**91.79 ± 1.58**

Furthermore, the complexity comparison in Table 9 indicates that SSA-MTSNet provides a reasonable balance between model capacity and computational efficiency. While the proposed model has a larger parameter count (~ 5.18 M) than most lightweight baselines, it is still substantially smaller than heavy Transformer-based models such as Bi-ViTNet (~ 170.00 M). Meanwhile, SSA-MTSNet attains the lowest inference latency (~ 0.40 ms/sample), demonstrating that the increased parameter scale does not compromise deployment efficiency. These results suggest that SSA-MTSNet offers a favorable trade-off among accuracy, stability, and inference efficiency.

**Table 9:** Comparison of model complexity and inference latency among SSA-MTSNet and baseline methods, where the baseline values are taken from the original publications or estimated from the reported architectures and experimental settings when exact values were unavailable

	Parameter (M)	Inference latency (ms/sample)
4D-aNN [53]	~ 0.74	~ 2.00
MDGCN-SRCNN [54]	~ 0.13	~ 3.00
DBGC-AFFNet-AFTL [55]	~ 0.24	~ 0.50
ACTNN [56]	~ 0.18	~ 0.50
Bi-ViTNet [57]	~ 170.00	~ 3.00
AMDET [58]	~ 0.30	~ 0.80
SSA-MTSNet (This work)	~ 5.18	~ 0.40

Beyond evaluating classification performance, the present findings also provide interpretable insights from both neuroscience and application perspectives. The attention analysis indicates that the proposed SSA-MTSNet can emphasize neurophysiologically meaningful spatial patterns, rather than relying on arbitrary electrode combinations. Furthermore, integrating multi-temporal scale convolution and LSTM enables the model to capture both short-term temporal variations and longer-range emotional dynamics. This is particularly important because emotional processing evolves over time rather than occurring as an instantaneous neural event.

### Performance analysis on the DEAP dataset

To comprehensively evaluate the generalization ability of the proposed model, we further evaluated the performance of SSA-MTSNet on the DEAP dataset, whose details have been described in Sect. 2.1. It also widely used dataset for EEG-based emotion recognition. Unlike SEED and SEED-IV, which are based on movie clips and target discrete emotion categories, DEAP adopts a different experimental paradigm and label definition. Specifically, DEAP uses music videos to elicit affective responses, and its classification tasks are typically defined in terms of the valence and arousal dimensions.

For the DEAP dataset, the model was trained under the corresponding experimental setting. After applying the same preprocessing procedure, 800 samples were obtained for each subject. To accommodate the different task settings, the batch size and learning rate were adjusted to 32 and 0.0003, respectively, while the other training hyperparameters remained the same as those used for the SEED and SEED-IV experiments.

As shown in Table 10, SSA-MTSNet achieves consistently strong performance across the SEED, SEED-IV, and DEAP datasets. Specifically, the proposed model achieves an accuracy of 98.34% ± 1.67% on SEED and 91.79% ± 1.58% on SEED-IV, demonstrating its effectiveness on datasets with discrete emotion categories. On the DEAP dataset, SSA-MTSNet further achieves 95.13% ± 0.67% for valence classification and 95.30% ± 0.51% for arousal classification. These results indicate that SSA-MTSNet also maintains competitive performance on DEAP, despite differences in electrode montage, data acquisition paradigms, emotional labeling strategies, and classification objectives.

**Table 10:** Generalization performance on the SEED, SEED-IV, and DEAP datasets

	SEED	SEED-IV	DEAP- valence	DEAP- arousal
	Acc ± Std(%)	Acc ± Std(%)	Acc ± Std(%)	Acc ± Std(%)
SSA-MTSNet	98.34 ± 1.67	91.79 ± 1.58	95.13 ± 0.67	95.30 ± 0.51

Overall, these findings suggest that the proposed architecture can adapt to datasets with different experimental paradigms and labeling strategies when trained under dataset-specific settings, thereby further supporting the robustness and generalizability of SSA-MTSNet for EEG-based emotion recognition.

### Limitations

The proposed model was evaluated on public datasets under offline experimental settings. Although subject-level tenfold cross-validation was adopted to mitigate subject leakage, the evaluation was still performed within the same datasets. Under these conditions, SSA-MTSNet showed competitive accuracy, relatively stable performance, and favorable inference efficiency on the tested platform, indicating its value as a promising framework for EEG-based emotion recognition research. However, its practical feasibility for resource-constrained devices, online systems, and broader affective computing applications remains to be further examined. Future work should therefore focus on external validation, cross-dataset evaluation, lightweight optimization, and more rigorous interpretability analysis, so that the methodological feasibility observed in this study can be translated into stronger practical feasibility.

## Conclusion

 In this paper, we proposed SSA-MTSNet, a multi-temporal scale EEG emotion recognition framework that combines serial spectral-spatial attention, spatio-temporal convolution, and LSTM-based temporal aggregation. Extensive experiments on SEED, SEED-IV, and DEAP demonstrate the superior performance of SSA-MTSNet. Specifically, the proposed model achieves 98.34% (Std = 1.67%) on SEED and 91.79% (Std = 1.58%) on SEED-IV, and 95.13% (Std = 0.67%) and 95.30% (Std = 0.51%) on the valence and arousal classification tasks of DEAP, respectively. The ablation results further suggest that the serial spectral-to-spatial attention design and the progressive multi-scale fusion strategy contribute to the overall performance of the model. Therefore, SSA-MTSNet is a competitive and promising architecture for EEG-based emotion recognition. Future work will focus on external validation, cross-dataset transfer evaluation, lightweight optimization, and more rigorous interpretability analysis.

## Data Availability

The datasets SEED and SEED-IV used in this study are publicly available at https://bcmi.sjtu.edu.cn/~seed/index.html. The DEAP dataset is available at http://www.eecs.qmul.ac.uk/mmv/data sets/deap/.

## References

[1] Pei G, Li H, Lu Y, Wang Y, Hua S, Li T (2024) Affective computing: recent advances, challenges, and future trends. Intell Comput 3:0076

[2] MacKenzie IS (2024) Human-computer interaction: an empirical research perspective

[3] Li X, Zhang Y, Tiwari P, Song D, Hu B, Yang M, Zhao Z, Kumar N, Marttinen P (2022) EEG based emotion recognition: a tutorial and review. ACM Comput Surv 55(4):1–57

[4] Pan J, Xie Q, Huang H, He Y, Sun Y, Yu R, Li Y (2018) Emotion-related consciousness detection in patients with disorders of consciousness through an EEG-based BCI system. Front Hum Neurosci 12:198 29867421 10.3389/fnhum.2018.00198 PMC5962793

[5] Yang D, Shin YI, Hong KS (2021) Systemic review on transcranial electrical stimulation parameters and EEG/fNIRS features for brain diseases. Front Neurosci 15:629323 33841079 10.3389/fnins.2021.629323 PMC8032955

[6] Dini H, Ghassemi F, Sendi MSE (2020) Investigation of brain functional networks in children suffering from attention deficit hyperactivity disorder. Brain Topogr 33(6):733–750 32918647 10.1007/s10548-020-00794-1

[7] Chang H, Zong Y, Zheng W, Tang C, Zhu J, Li X (2022) Depression assessment method: an EEG emotion recognition framework based on spatiotemporal neural network. Front Psychiatry 12:837149 35368726 10.3389/fpsyt.2021.837149 PMC8967371

[8] Li X, Huang G, Liu Y, Hong KS (2026) EEG-based depression detection using a local-global feature fusion deep learning network. Biomed Signal Process Control 118:109681

[9] Hu H, Zhu Z, Gao Z, Zheng R (2018) Analysis on biosignal characteristics to evaluate road rage of younger drivers: A driving simulator study. In: Proc 2018 IEEE intelligent vehicles symposium (IV), pp 156–161

[10] Yang D, Huang R, Yoo SH, Shin MJ, Yoon JA, Shin YI, Hong KS (2020) Detection of mild cognitive impairment using convolutional neural network: temporal-feature maps of functional near-infrared spectroscopy. Front Aging Neurosci 12:141 32508627 10.3389/fnagi.2020.00141 PMC7253632

[11] Yoo SH, Woo SW, Shin MJ, Yoon JA, Shin YI, Hong KS (2020) Diagnosis of mild cognitive impairment using cognitive tasks: a functional near-infrared spectroscopy study. Curr Alzheimer Res 17(13):1145–1160 33583382 10.2174/1567205018666210212154941

[12] Kang MK, Hong KS, Yang D, Kim HK (2025) Multi-scale neural networks classification of mild cognitive impairment using functional near-infrared spectroscopy. Biocybern Biomed Eng 45(1):11–22

[13] Wang Z, Huang G, Chen Z, Liu X, Liu Y, Hong KS (2026) Decoupled bidirectional spatio-temporal fusion network for hybrid EEG-fNIRS cognitive task classification. Brain Sci 16(2):241 41750241 10.3390/brainsci16020241 PMC12938437

[14] Adolphs R, Anderson DJ (2018) The neuroscience of emotion: a new synthesis. Princeton University Press, Princeton, NJ, USA

[15] Nguyen HD, Kim SH, Lee GS, Yang HJ, Na IS, Kim SH (2022) Facial expression recognition using a temporal ensemble of multi-level convolutional neural networks. IEEE Trans Affect Comput 13(1):226–237

[16] Noroozi F, Corneanu CA, Kamińska D, Sapiński T, Escalera S, Anbarjafari G (2021) Survey on emotional body gesture recognition. IEEE Trans Affect Comput 12(2):505–523

[17] Li R, Yang D, Fang F, Hong KS, Reiss AL, Zhang Y (2022) Concurrent fNIRS and EEG for brain function investigation: a systematic, methodology-focused review. Sensors Basel 22(15):5865 35957421 10.3390/s22155865 PMC9371171

[18] Khan MJ, Ghafoor U, Hong KS (2018) Early detection of hemodynamic responses using EEG: a hybrid EEG-fNIRS study. Front Hum Neurosci 12:479. 10.3389/fnhum.2018.00479 30555313 10.3389/fnhum.2018.00479 PMC6281984

[19] Yang D, Kang MK, Huang G, Eggebrecht AT, Hong KS (2024) Repetitive transcranial alternating current stimulation to improve working memory: an EEG-fNIRS study. IEEE Trans Neural Syst Rehabil Eng 32:1257–1266 38498739 10.1109/TNSRE.2024.3377138

[20] Li W, Zhang Z, Song A (2021) Physiological signal-based emotion recognition: an odyssey from methodology to philosophy. Measurement (Lond) 172:108747

[21] Morawetz C, Bode S, Baudewig J, Heekeren HR (2017) Effective amygdala-prefrontal connectivity predicts individual differences in successful emotion regulation. Soc Cogn Affect Neurosci 12(4):569–585 27998996 10.1093/scan/nsw169 PMC5390747

[22] Berboth S, Morawetz C (2021) Amygdala-prefrontal connectivity during emotion regulation: a meta-analysis of psychophysiological interactions. Neuropsychologia 153:107767 33516732 10.1016/j.neuropsychologia.2021.107767

[23] Hong KS, Khan MJ (2017) Hybrid brain-computer interface techniques for improved classification accuracy and increased number of commands: a review. Front Neurorobot 11:35 28790910 10.3389/fnbot.2017.00035 PMC5522881

[24] Hong KS, Khan MJ, Hong MJ (2018) Feature extraction and classification methods for hybrid fNIRS-EEG brain-computer interfaces. Front Hum Neurosci 12:246 30002623 10.3389/fnhum.2018.00246 PMC6032997

[25] Petrantonakis PC, Hadjileontiadis LJ (2010) Emotion recognition from EEG using higher order crossings. IEEE Trans Inf Technol Biomed 14(2):186–197 19858033 10.1109/TITB.2009.2034649

[26] Mehmood RM, Bilal M, Vimal S, Lee SW (2022) EEG-based affective state recognition from human brain signals by using Hjorth-activity. Measurement 202:11173

[27] Alsolamy M, Fattouh A (2016) Emotion estimation from EEG signals during listening to Quran using PSD features. In: Proc 2016 7th international conference on computer science and information technology (CSIT), pp 1–5

[28] Mohammadi Z, Frounchi J, Amiri M (2017) Wavelet-based emotion recognition system using EEG signal. Neural Comput Appl 28(8):1985–1990

[29] Chen T, Ju S, Yuan X, Elhoseny M, Ren F, Fan M, Chen Z (2018) Emotion recognition using empirical mode decomposition and approximation entropy. Comput Electr Eng 72:383–392

[30] Jie X, Cao R, Li L (2014) Emotion recognition based on the sample entropy of EEG. Biomed Mater Eng 24(1):1185–1192 24212012 10.3233/BME-130919

[31] Lan Z, Sourina O, Wang L, Liu Y (2016) Real-time EEG-based emotion monitoring using stable features. Vis Comput 32(3):347–358

[32] Duan RN, Zhu JY, Lu BL (2013) Differential entropy feature for EEG-based emotion classification. In: Proc 2013 6th international IEEE/EMBS conference on neural engineering (NER), pp 81–84. 10.1109/NER.2013.6695876

[33] Chung SY, Yoon HJ (2012) Affective classification using Bayesian classifier and supervised learning. In: Proc 2012 12th international conference on control, automation and systems, pp 1768–1771

[34] Ackermann P, Kohlschein C, Bitsch JA, Wehrle K, Jeschke S (2016) EEG-based automatic emotion recognition: Feature extraction, selection and classification methods. In: Proc 2016 IEEE 18th international conference on e-health networking, applications and services (Healthcom), pp 1–6

[35] Bhatti AM, Majid M, Anwar SM, Khan B (2016) Human emotion recognition and analysis in response to audio music using brain signals. Comput Human Behav 65:267–275

[36] Murugappan M, Rizon M, Nagarajan R, Yaacob S, Zunaidi I, Hazry D (2008) Lifting scheme for human emotion recognition using EEG. In: Proc 2008 international symposium on information technology, vol 2, pp 1–7

[37] Zheng WL, Lu BL (2015) Investigating critical frequency bands and channels for EEG-based emotion recognition with deep neural networks. IEEE Trans Auton Ment Dev 7(3):162–175

[38] Yang YX, Gao ZK, Wang XM, Li YL, Han JW, Marwan N, Kurths J (2018) A recurrence quantification analysis-based channel-frequency convolutional neural network for emotion recognition from EEG. Chaos 28(8):085724 30180618 10.1063/1.5023857

[39] Li J, Zhang Z, He H (2018) Hierarchical convolutional neural networks for EEG-based emotion recognition. Cognit Comput 10(2):368–380

[40] Song T, Zheng W, Song P, Cui Z (2020) EEG emotion recognition using dynamical graph convolutional neural networks. IEEE Trans Affect Comput 11(3):532–541

[41] Greiner G, Zhang Y (2024) Multi-modal EEG NEO-FFI with trained attention layer (MENTAL) for mental disorder prediction. Brain Inf 11:26 10.1186/s40708-024-00240-z PMC11496460 39436529

[42] Islam M, Lee T (2025) An automated extraction of spectral-temporal and spatial-temporal features of EEG for emotion detection. Brain Inf 12(1):1–19 10.1186/s40708-025-00265-y PMC12317964 40751855

[43] Patel P, Balasubramanian S, Annavarapu RN (2024) Cross subject emotion identification from multichannel EEG sub-bands using Tsallis entropy feature and KNN classifier. Brain Inf 11:7 10.1186/s40708-024-00220-3 PMC11358557 38441825

[44] Dong Y, Jing C, Mahmud M, Ng MKP, Wang SQ (2024) Enhancing cross-subject emotion recognition precision through unimodal EEG: a novel emotion preceptor model. Brain Inf 11:31 10.1186/s40708-024-00245-8 PMC11655793 39692977

[45] Li R, Yang X, Lou J, Zhang J (2024) A temporal-spectral graph convolutional neural network model for EEG emotion recognition within and across subjects. Brain Inf 11:30 10.1186/s40708-024-00242-x PMC11655824 39692964

[46] Zheng WL, Liu W, Lu Y, Lu BL, Cichocki A (2019) Emotionmeter: a multimodal framework for recognizing human emotions. IEEE Trans Cybern 49(3):1110–1122 29994384 10.1109/TCYB.2018.2797176

[47] Koelstra S, Muhl C, Soleymani M, Lee JS, Yazdani A, Ebrahimi T, Pun T, Nijholt A, Patras I (2011) DEAP: a database for emotion analysis using physiological signals. IEEE Trans Affect Comput 3(1):18–31

[48] Woo S, Park J, Lee JY, Kweon IS (2018) CBAM: Convolutional block attention module. In: Proc European conference on computer vision (ECCV), pp 3–19

[49] Reznik SJ, Allen JJB (2018) Frontal asymmetry as a mediator and moderator of emotion: an updated review. Psychophysiology 55(1):12965 10.1111/psyp.12965 28776710

[50] Seo D, Olman CA, Haut KM, Sinha R, MacDonald IAW, Patrick CJ (2014) Neural correlates of preparatory and regulatory control over positive and negative emotion. Soc Cogn Affect Neurosci 9(4):494–504 23887812 10.1093/scan/nst115 PMC3989138

[51] Hadjidimitriou SK, Hadjileontiadis LJ (2012) Toward an EEG-based recognition of music liking using time-frequency analysis. IEEE Trans Biomed Eng 59(12):3498–3510 23033323 10.1109/TBME.2012.2217495

[52] Jenke R, Peer A, Buss M (2014) Feature extraction and selection for emotion recognition from EEG. IEEE Trans Affect Comput 5(3):327–339

[53] Xiao G, Shi M, Ye M, Xu B, Chen Z, Ren Q (2022) 4d attention-based neural network for EEG emotion recognition. Cogn Neurodyn 16(4):805–818 35847538 10.1007/s11571-021-09751-5 PMC9279544

[54] Bao G, Yang K, Tong L, Shu J, Zhang R, Wang L, Yan B, Zeng Y (2022) Linking multi-layer dynamical GCN with style-based recalibration CNN for EEG-based emotion recognition. Front Neurorobot 16:834952 35280845 10.3389/fnbot.2022.834952 PMC8907537

[55] Sun M, Cui W, Yu S, Han H, Hu B, Li Y (2022) A dual-branch dynamic graph convolution based adaptive transformer feature fusion network for EEG emotion recognition. IEEE Trans Affect Comput 13(4):2218–2228

[56] Gong L, Li M, Zhang T, Chen W (2023) EEG emotion recognition using attention-based convolutional transformer neural network. Biomed Signal Process Control 84:104835

[57] Lu W, Tan TP, Ma H (2023) Bi-branch vision transformer network for EEG emotion recognition. IEEE Access 11:36233–36243

[58] Xu Y, Du Y, Li L, Lai H, Zou J, Zhou T, Xiao L, Liu L, Ma P (2024) AMDET: attention based multiple dimensions EEG transformer for emotion recognition. IEEE Trans Affect Comput 15(3):1067–1077

